# Abated crowding by fast-tracking the Throughput component of the ED for patients in no need of hospitalization with competency managed personnel

**DOI:** 10.1186/s12873-024-01069-9

**Published:** 2024-08-15

**Authors:** Jesper Juul Larsen, Halfdan Lauridsen, Laurits Wullum Gundersen, Birgit Falk Riecke, Thomas A. Schmidt

**Affiliations:** 1 Department of Emergency Medicine, North Zealand Hospital, Hillerød, Denmark; 2 Department of Digitalization and Analysis, North Zealand Hospital, Hillerød, Denmark; 3 Institute of Clinical Medicine, Faculty of Health and Medical Sciences, University of Copenhagen, Copenhagen, Denmark

**Keywords:** Crowding, Emergency Department, SAP Web Intelligence tool, Emergency outpatient incident registration (EOIR), Electronic health record

## Abstract

**Background:**

Emergency department (ED) crowding is a major patient safety concern and has a negative impact on healthcare systems and healthcare providers. We hypothesized that it would be feasible to control crowding by employing a multifaceted approach consisting of systematically fast-tracking patients who are mostly not in need of a hospital stay as assessed by an initial nurse and treated by decision competent physicians.

**Methods:**

Data from 120,901 patients registered in a secondary care ED from the 4t^th^ quarter of 2021 to the 1st quarter of 2024 was drawn from the electronic health record’s data warehouse using the SAP Web Intelligence tool and processed in the Python programming language. Crowding was compared before and after ED transformation from a uniform department into a high flow (α) and a low flow (β) section with patient placement in gurneys/chairs or beds, respectively. Patients putatively not in need of hospitalization were identified by nurse, placed in in the α setting and assessed and treated by decision competent physicians. Incidence of crowding, number of patients admitted per day and readmittances within 72 h following ED admission before and after changes were determined. Values are number of patients, mean ± SEM and mean differences with 95% CIs. Statistical significance was ascertained using Student’s two tailed t-test for unpaired values.

**Results:**

Before and after ED changes crowding of 130% amounted to 123.8 h and 19.3 h in the latter. This is a difference of -104.6 ± 23.9 h with a 95% CI of -159.9 to -49.3, Δ% -84 (*p* = 0.002).

There was the same amount of patients / day amounting to 135.8 and 133.5 patients / day Δ% = -1.7 patients 95% CI -6.3 to 1.6 (*p* = 0.21).

There was no change in readmittances within 72 h before and after changes amounting to 9.0% versus 9.5%, Δ% = 0.5, 95%, CI -0.007 to 1.0 (*p* > 0.052).

**Conclusion:**

It appears feasible to abate crowding with unchanged patient admission and without an increase in readmittances by fast-track assessment and treatment of patients who are not in need of hospitalization.

## Background

Emergency Department (ED) crowding is defined as a situation where the demand for emergency services exceeds the ability of an emergency department (ED) to provide quality care within appropriate time frames [1] and bedevils Eds worldwide.

The current paper is a single center digital evaluation of crowding carried out at the Emergency Department (ED) at North Zealand hospital situated in the Capital Region of Denmark. Its population base is approx. 317,000 citizens.

The ED at North Zealand Hospital handles the treatment of all acute patients ≥ 18 years of age referred for evaluation except for gynecological/obstetric patients. Approx. 50.000 referred citizens per year are treated in the ED. The ED has its own staff of nurses and physicians.

Crowding has been a relentless problem in our ED estimated to be between 130 – 150% above capacity quarterly.

Crowding is a major patient safety concern and has a large impact on the healthcare system and healthcare providers’ wellbeing. Understanding the magnitude of these effects should motivate the development of interventions to better control crowding in EDs globally [2, 3].

The most commonly cited factor, and the most important in terms of patient care, is that ED crowding increases the chances of adverse events and mortality for patients [4].

We hypothesized that it would be feasible to control crowding by employing a multifaceted approach consisting of systematically fast-tracking patients who are mostly not in need of a hospital stay, because such patients make up a substantial presence in the ED to no critical avail.

## Methods

**Fig. 1:**
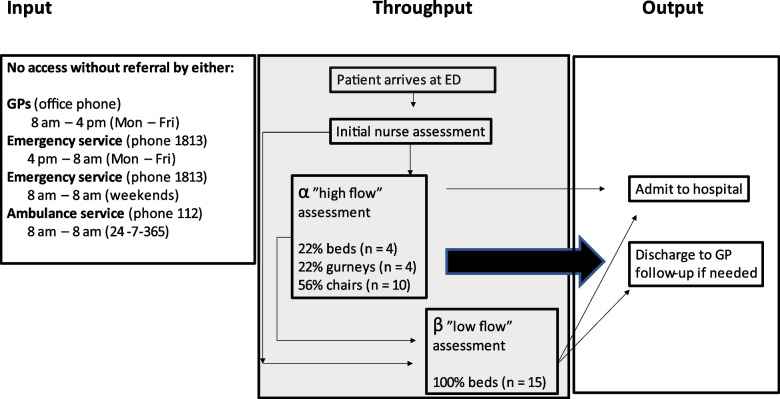
An illustration of Input, Throughput and Output of the ED following 2nd quarter of 2023 after which the Outflow component had been facilitated by competency management of the initial nurse and physicians in the Throughput component with placement of patients mostly limited to gurneys and chairs in a high flow section (α) of the ED. GP = general practitioner

To test the hypothesis and to contribute to the field related to ED crowding, the department was divided into a high flow (α) and a low flow (β) section as of the second quarter of 2023 (Fig. 1).

In the high flow α section hospital beds were replaced predominantly by gurneys and chairs without increasing the amount of patient contact points. Thua, in the high flow α section hospital beds were limited to comprise only 22% (*n* = 4) of possible patient occupancy reserved for emergent cases, while gurneys comprise another 22% (*n* = 4) reserved for less affected patients; and chairs make up 56% (*n* = 10) for ambulatory patients.

In the low flow β section hospital beds comprised 100% (*n* = 15) of possible patient occupancy. Patients in the β section were usually in need of admission to a hospital stay.

All included patients were referred for evaluation regarding possible admission to hospital because of suspicion of an internal medicine or surgical ailment i.e., stroke, appendicitis, myocardial infarction, pneumonia, and other illnesses commonly requiring in-hospital treatment. The population did not include patients with minor casualties or patients referred for out of hours general practitioner service, who are not assessed in the ED proper.

We created a different attendance profile for the staff so that there was a better match between the staff's competences and the patients' complexity.

Competent initial nurses and α section physicians were selected by the head nurses and the chief physician respectfully following performance evaluation based on performance and the premise that competency is a function of aptitude and expeditiousness. By careful planning it was ensured that the necessary competencies were duly available on each day shift. There were no other rearrangements of staff nor more physicians allocated.

Because all patients were referred by a general practitioner or a Medical Helpline and a referral note was stated on electronic screens, the initial nurse expected and could assess, and place patients as deemed appropriate upon arrival or following a waiting room stay.

Admission of patients not in need of hospitalization to fast track assessment was performed by the initial nurse, if requested in collaboration with a senior α section physician, based upon cause of admission note and clinical eyeballing. All other patients were duly triaged using the Danish Emergency Process Triage (DEPT) and patients ≥ 65 years of age were also frailty scored (Clinical Frailty Scale).

Furthermore, all patients had an initial set of blood samples drawn upon arrival to facilitate fast diagnostic decision making. Patients were upon arrival placed adjacent to the blood sampling facility instead of in the waiting-room until blood samples were drawn. Triage level was allocated wherever the patient was located.

Data were drawn from the electronic health record (EHR) system Epic’s data warehouse using the SAP Web Intelligence tool [5] and processed in the Python programming language (with the Anaconda distribution) [6].

The course of every patient admitted to the ED was registered using specific Emergency Outpatient Incident Registration (EOIR) that include timestamps for arrival and departure as well as every placement of a patient in the ED.

The basic population was defined as having had an EOIR in the ED in the period in question.

An emergency course was defined as a continuous period during which the patient was physically present in the ED. The start and end times for a course were therefore the respective first and last EOIR before the patient either was discharged or moved to another treatment facility.

A stay in the waiting room was defined at the initial period from the course start time until the patient was first registered as having been placed relevantly in the α and β sections of the ED.

The data was aggregated, i.e., it was counted how many patients had an active course every whole minute throughout the ED including hallways and the waiting room.

Finally, the maximum number of patients present at the same time per hour and day was counted. There were 33 physical gurneys, chairs, or beds in the department. Thus when 33 patients were registered in the ED (waiting room, hallway, gurneys, chairs, or beds) the occupancy rate was 100%.

### Statistics

Values are given as numbers of patients and means ± SEM. Statistical significance was ascertained by Student’s two-tailed t-test for unpaired observations before and after implementation of fast-tracking patients who were deemed not in need of a hospital stay as assessed by an initial nurse and treated by decision competent physicians. Statistical significances are assessed using confidence intervals and *p* values < 0.05.

## Results

**Fig.2:**
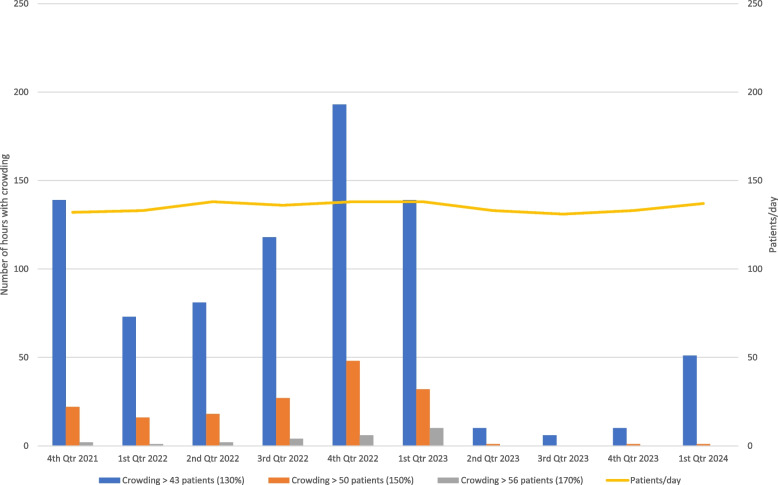
An illustration of the number of hours with crowding amounting to > 130% (blue columns), crowding amounting to > 150% (orange columns) and crowding amounting to > 170% (grey columns) from the 4th quarter of 2021 to 1st quarter of 2024. Note that the 3rd quarter of 2021 ended the COVID area and is not included in calculations. The yellow line indicates the number of patients in the ED per day. Fast-tracking the outflow component of the Emergency Department by competency management of the initial nurse and physicians including placement of patients mostly limited to gurneys and chairs was initiated 2nd quarter of 2023

The study period was from October 1. 2021 (3rd quarter of 2021) until March 31, 2024. comprising 120,901 patients (Fig. 2).

Following the lifting of COVID restrictions in the 3rd quarter of 2021 and subsequent ordinary use of the waiting room without separation, because > 90% of the adult Danish population was vaccinated, the ED had a serious crowding problem with crowding exceeding 130% from 73 to 193 h per quarter. For the most crowded quarter it amounted to 8.8% of the time. Crowding exceeded 150% from 16 to 48 h per quarter, which amounted to 2,2% of the time in the most crowded quarter. Crowding exceeded 170% from 1 to 10 h per quarter.

Following implementation of the described fast-track system, in the 2nd quarter of 2023, crowding exceeding 130% decreased to 6 h –51 h per quarter, that is at most 2% of the time. In total there was only 1 h of crowding exceeding 150% and 0 h of crowding exceeding 170%.

Comparing crowding of 130% before (4th quarter of 2021 to the 1st quarter of 2023) and after (2nd quarter of 2023 to the 1st quarter of 2024) implementation of fast-tracking it amounted to a mean value per quarter of 123.8 h in the former and 19.3 h in the latter. This is a difference of -104.6 ± 23.9 h with a 95% CI of -159.9 to -49.3, Δ% -84 (*p* = 0.002).

It should be observed (Fig. 2) that before (4th quarter of 2021 to the 1st quarter of 2023) and after (2nd quarter of 2023 to the 1st quarter of 2024) implementation of the fast-track system, there was the same amount of patients/ day amounting to a mean value of 135.8 and 133.5 patients / day with only a difference of –2.3 ± 1.7 patients 95%CI -6.3 to 1.6 (*p* > 0.052).

Using data from the last quarter of 2022 before ED changes compared with the last quarter of 2023 after ED changes, it took 4.9 effective h to evaluate 37 patients per day before our multifaceted approach. Following ED change, it took 3.9 effective h to evaluate 50 patients per day in the same patient positions. Thus, following ED change it requires 1 effective h less to evaluate 35% more patients. This cannot be considered the overall patient turn over effect of ED changes but an estimate of comparable annual quarters.

**Fig. 3:**
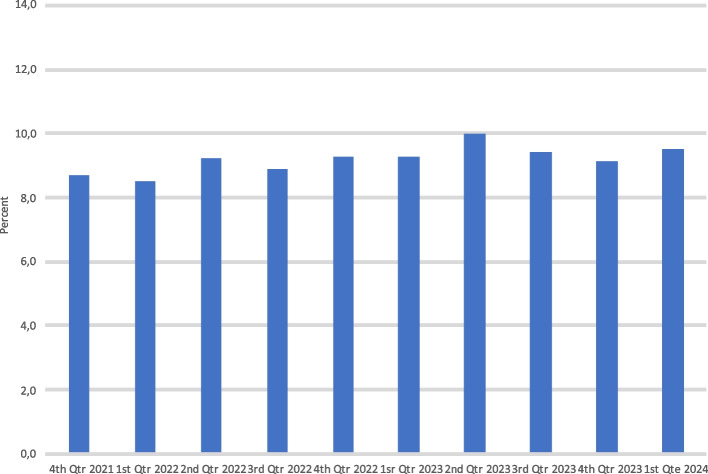
Readmittances within 72 h following ED admission from 4th quarter 2021 till 1st quarter 2024. No significant impact is seen on readmittances following the establishment of a high flow section with mostly gurneys and chairs as a placement possibility

Figure 3 shows the level of readmittances within 72 h in percent. There was a non-significant difference between the two periods amounting to a mean value of 9.0% versus 9.5% readmittances amounting to an absolute difference of 0.5% 95% CI -0.007 to 1.0 (*p* = 0.52).

## Discussion

### Principal findings

The conceptual model of crowding partitions ED crowding into 3 interdependent components: Input, Throughput, and Output. These components often exist within an acute care system that is characterized by the delivery of unscheduled care [7]. However, this is not the case in the current study, because in the Capital Region of Denmark (Fig. 1) all patients in need of emergency care are referred either via patients’ general practitioner, the ambulance service or a general emergency service by phone [8], but mandatory referral does not eliminate crowding (Fig. 2).

The Throughput-factor on the other hand is a different matter, because it relies solely on what happens in the ED. We therefore decided to qualify and minimize stay of patients in the Throughput component (Fig. 1). to optimize the output component which we have found to be feasible and efficient.

By this approach crowding > 130% was reduced by 104.6 ± 23.9 h with a 95% CI of -159.9 to -49.3, Δ% -84 (*p* = 0.002). This reduction was achieved with the same patient intake of 135.8 and 133.5 patients / day Δ% = -1.7 patients 95% CI -6.3 to 1.6 (*p* = 0.21) Furthermore, it did not increase readmittances within 72 h before and after changes amounting to a mean value of 9.0% versus 9.5%, Δ% = 0.5, 95%, CI -0.007 to 1.0 (*p* > 0.052).

Other departments have experimented with elements of our approach with a varying degree of success [9]. Our study showed that it is important to establish several, if not all, of the described initiatives, because implementation of e.g., chairs alone make no difference. Patients not in need of hospitalization, i.e., the right patients must be selected to be put in the chairs by initial nurse assessment. A decision competent medical doctor must be allocated to fast-track medical evaluation and treatment based on focused training and assessment. Standard blood tests must be quickly available, i.e., frontloading of blood tests. Also, a less expeditious track needs to be available for the patients who are forecasted to need admission to a hospital bed but should of course also be manned by competent staff.

### Strengths and weaknesses of the study

It is a strength that the patient volume in our study is large, i.e., 120,901 patients.

It is a strength that the course of every patient admitted to the ED was registered using specific EOIR that among other things include timestamps for arrival and departure as well as every placement of a patient in the ED. It is also a strength that a stay in the waiting room was registered and aggregated because it ensured inclusion of all patients present in the ED.

Conventionally it is considered a weakness that the study is a single center study. It would be interesting to extend the setup to other Eds and do a multicenter study. On the other hand solutions to ED crowding will be unique and require tailoring to local circumstances [3], thus it does not seem readily possible to harvest data from several Eds without consent to organizational and logistic changes.

It is unanswered whether the marked abatement seen in crowding is sustainable. This question needs to be followed up by future research.

## Conclusions

Crowding may be successfully controlled by employing a multifaceted approach mainly consisting of systematically fast-tracking patients who are mostly not in need of a hospital stay as assessed by an initial nurse and treated by decision competent physicians.

Most recent publications are analysis studies and reviews [3, 10] that focus on the many facets of crowding. However, to our knowledge the current study is the first study of crowding based on novel data that presents a solution to the problem.

## Data Availability

The datasets used and / or analyzed during the current study are available from the corresponding author on reasonable request.

## Abbreviations

ED: Emergency department
HER: Electronic health record
EOIR: Emergency outpatient incident registration
SEM: Standard error of the mean
CI: Confidence interval

## References

[1] Affleck A, Parks P, Drummond A, Rowe BH, Ovens HJ. Emergency department overcrowding and access block. CJEM. 2013;15(6):359–84. 24176460 10.1017/S1481803500002451

[2] Pearce S, Marr E, Shannon T, Marchand T, Lang E. Overcrowding in emergency departments: an overview of reviews describing global solutions and their outcomes. Intern Emerg Med. 2024;19(2):483–91. 38041766 10.1007/s11739-023-03477-4

[3] Pearce S, Marchand T, Shannon T, Ganshorn H, Lang E. Emergency department crowding: an overview of reviews describing measures causes, and harms. Intern Emerg Med. 2023;18(4):1137–58. 36854999 10.1007/s11739-023-03239-2 PMC9974385

[4] Jones S, Moulton C, Swift S, Molyneux P, Black S, Mason N, et al. Association between delays to patient admission from the emergency department and all-cause 30-day mortality. Emerg Med J. 2022;39(3):168–73. 35042695 10.1136/emermed-2021-211572

[5] SAP BusinessObjects Web Intelligence [Internet]. Available from: https://help.sap.com/docs/SAP_BUSINESSOBJECTS_WEB_INTELLIGENCE?locale=en-US.

[6] Anaconda distribution [Internet]. Available from: https://www.anaconda.com/download.

[7] Asplin BR, Magid DJ, Rhodes KV, Solberg LI, Lurie N, Camargo CA. A conceptual model of emergency department crowding. Ann Emerg Med. 2003;42(2):173–80. 12883504 10.1067/mem.2003.302

[8] Emergency Medical Services 1813 [Internet]. Available from: https://www.regionh.dk/english/Healthcare-Services/Emergency-Medical-Services/Pages/default.aspx.

[9] Hesselink G, Sir Ö, Schoon Y. Effectiveness of interventions to alleviate emergency department crowding by older adults: a systematic review. BMC Emerg Med. 2019;19(1):69. 31747917 10.1186/s12873-019-0288-4 PMC6864956

[10] Kremers MNT, Nanayakkara PWB, Levi M, Bell D, Haak HR. Strengths and weaknesses of the acute care systems in the United Kingdom and the Netherlands: what can we learn from each other? BMC Emerg Med. 2019;19(1):40. 31349797 10.1186/s12873-019-0257-y PMC6660652

[11] The Danish Health Care Act [Internet]. Available from: https://www.retsinformation.dk/eli/lta/2024/247.

